# Neurophysiological characterization of stroke recovery: A longitudinal TMS and EEG study

**DOI:** 10.1111/cns.14471

**Published:** 2023-09-18

**Authors:** Qian Ding, Jixiang Chen, Shunxi Zhang, Songbin Chen, Xiaotong Li, Yuan Peng, Yujie Chen, Junhui Chen, Kang Chen, Guiyuan Cai, Guangqing Xu, Yue Lan

**Affiliations:** ^1^ Department of Rehabilitation Medicine, Guangzhou First People's Hospital South China University of Technology Guangzhou China; ^2^ Department of Rehabilitation Medicine, Guangdong Provincial People's Hospital (Guangdong Academy of Medical Sciences) Southern Medical University Guangzhou China; ^3^ Guangzhou Key Laboratory of Aging Frailty and Neurorehabilitation Guangzhou China

**Keywords:** EEG, functional connectivity, stroke recovery, TMS, TMS‐EEG

## Abstract

**Aims:**

Understanding the neural mechanisms underlying stroke recovery is critical to determine effective interventions for stroke rehabilitation. This study aims to systematically explore how recovery mechanisms post‐stroke differ between individuals with different levels of functional integrity of the ipsilesional corticomotor pathway and motor function.

**Methods:**

Eighty‐one stroke survivors and 15 age‐matched healthy adults participated in this study. We used transcranial magnetic stimulation (TMS), electroencephalography (EEG), and concurrent TMS‐EEG to investigate longitudinal neurophysiological changes post‐stroke, and their relationship with behavioral changes. Subgroup analysis was performed based on the presence of paretic motor evoked potentials and motor function.

**Results:**

Functional connectivity was increased dramatically in low‐functioning individuals without elicitable motor evoked potentials (MEPs), which showed a positive effect on motor recovery. Functional connectivity was increased gradually in higher‐functioning individuals without elicitable MEP during stroke recovery and influence from the contralesional hemisphere played a key role in motor recovery. In individuals with elicitable MEPs, negative correlations between interhemispheric functional connectivity and motor function suggest that the influence from the contralesional hemisphere may be detrimental to motor recovery.

**Conclusion:**

Our results demonstrate prominent clinical implications for individualized stroke rehabilitation based on both functional integrity of the ipsilesional corticomotor pathway and motor function.

## Graphical abstract

Understanding the neural mechanisms underlying stroke recovery is critical to determine effective interventions for stroke rehabilitation. The contralesional hemisphere plays a supportive role in individuals with impaired corticomotor pathway, while plays a negative role in individuals with intact corticomotor pathway regardless of motor function. Individualized stroke rehabilitation should be developed based on both functional integrity of corticomotor pathway and motor function.

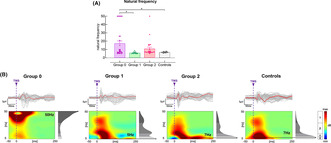

## 1 INTRODUCTION

Over half of stroke survivors are greatly disabled despite intensive rehabilitation, many with persistent motor symptoms impacting functional independence in daily life.^1^ Motor deficits post‐stroke are associated with altered cortical activity and dysfunctional brain networks.^2^ Various non‐invasive neuromodulation techniques have been used to promote normal brain functioning and facilitate motor recovery post‐stroke.

Typical neuromodulation approaches are developed using the interhemispheric competition model, which states that a stroke lesion disrupts the balance between hemispheres to cause excessive interhemispheric inhibition (IHI) from the contralesional hemisphere (CH) to the ipsilesional hemisphere (IH).^3^, ^4^ Nevertheless, some recent studies have suggested that the interhemispheric competition model may apply only to mildly impaired individuals, while for severely impaired individuals, CH may play an important role in motor recovery,^3^, ^4^ but there is still no direct evidence supporting this.^5^ There is a need to understand the factors influencing post‐stroke recovery mechanisms to inform individualized intervention protocols.

Factors such as functional integrity of the ipsilesional corticomotor pathway [usually indicated by the presence of motor evoked potentials (MEPs) of the affected arm], and motor function have been suggested to influence recovery mechanisms post‐stroke.^4^ There have been studies classifying stroke survivors based on motor scores^6^, ^7^ or functional integrity of the ipsilesional corticomotor pathway,^8^, ^9^ but there is a dearth of studies accounting for both factors, possibly due to the assumption that higher‐functioning individuals always have less impaired ipsilesional corticomotor pathway. However, for individuals in whom alternative motor pathways have taken the place of movement control in the paretic limb, the ipsilesional corticomotor pathway may not be intact even though they have relatively good motor function.^3^ Therefore, it is necessary to take both motor function and functional integrity of the ipsilesional corticomotor pathway into consideration.

Electroencephalography (EEG)^10^, ^11^, ^12^ and transcranial magnetic stimulation (TMS)^13^, ^14^ are commonly used non‐invasive techniques for studying brain functions. EEG signals can be used as a sensitive measure of brain functional connectivity (FC), reflecting the synchrony of neural activity in functionally cooperating but anatomically distinct brain regions.^2^ TMS can be used to elicit MEPs. The presence of MEPs suggests functional integrity of the ipsilesional corticomotor pathway, and therefore provides valuable prognostic information about functional outcome.^15^ Concurrent TMS and EEG (TMS‐EEG) can be used to measure cortical activity and oscillatory events simultaneously regardless of the integrity of corticospinal tracts.^16^ A combination use of multiple tools allows to investigate neural mechanisms of stroke functional recovery from different perspectives.^4^

Here, we used TMS, EEG, and TMS‐EEG to investigate neurophysiological changes post‐stroke and their relationships with behavioral changes. We performed subgroup analysis based on motor function and the presence of MEPs of the affected arm to compare recovery mechanisms of individuals with different clinical characteristics. We hypothesized that in low‐functioning MEP (−) individuals, FC from intact brain regions may benefit motor recovery; in higher‐functioning MEP (−) individuals, the influence from CH would play an important role in motor recovery; finally, in MEP (+) individuals, the influence from CH may hinder motor recovery post‐stroke.

## 2 METHODS

### 2.1 Subjects

Eighty‐one stroke and 15 age‐matched healthy adults participated in this study. This study involves human participants and was approved by Guangzhou First People's Hospital Human Research Ethics Committee (K‐2021‐130‐01). Participants gave informed consent to participate in the study before taking part. Stroke survivors were recruited if involved in a single stroke within 12 months prior to enrollment. Patients were excluded if they showed any cognitive impairment. All participants were screened for eligibility to receive TMS. TMS exclusion criteria were pregnancy, medications that modulate seizure threshold, or any metal or implanted devices. Participants were instructed to avoid alcohol or caffeine prior to the experiment.

### 2.2 Experimental procedures

In Session 1, 81 stroke survivors and 15 healthy adults underwent behavioral and neurophysiological measurements. All participants completed the power grip strength assessment. Stroke survivors also completed the upper‐extremity component of the Fugl‐Meyer motor function assessment (UE FMA) and action research arm test (ARAT), to evaluate motor deficit and upper limb motor function, respectively. Neurophysiological measures included TMS, EEG, and TMS‐EEG. 46 stroke survivors returned after ~2 months to undergo Session 2 measurements, which were the same as those in Session 1. In both sessions, neurophysiological measures were conducted after the behavioral measures.

### 2.3 Force measurements

Isometric pinch grip force in the “standard” position were measured using the grip force assessment system (BioFlex‐H, Zhanghe Intelligent Co.) coupled with real‐time force feedback.^17^ Three maximal voluntary isometric pinch grip (MVC) trials were separated by 2‐min rest intervals in both hands of each participant. The peak value was recorded as MVC for each hand.

### 2.4 Electroencephalography (EEG)

#### 2.4.1 EEG recordings

EEG signals were acquired using TMS‐compatible EEG cap (ANT Neuro) equipped with 64 Ag/AgCl electrodes in a layout operated by the extended international 10–20 system for electrode placement.^18^ All channels were referenced online to CPz and amplified with an EEGO amplifier (ANT Neuro). Sampling rate was set at 2048 Hz and impedances were maintained under 10 kΩ for all channels. During the 6‐min resting EEG recording, individuals sat comfortably in a dimly lit, sound‐shielded room with eyes closed.

#### 2.4.2 EEG analysis

Offline analyses of EEG signals were performed using MATLAB2019b (Mathworks, Inc.). EEG data were preprocessed with EEGLAB toolbox (version 14.1.2b).^19^ Custom MATLAB scripts were used for power and FC analyses. Interhemispheric coherence and phase locking value (PLV) were calculated indicating interhemispheric FC. Graph theory analysis was performed to measure global (including efficiency and small‐worldness) and local (degree centrality, betweenness centrality, and clustering coefficient) network connectivity.^20^, ^21^ Details see Data S1.

### 2.5 Transcranial Magnetic Stimulation (TMS)

#### 2.5.1 TMS recordings

TMS recordings were performed following behavioral measurement. TMS was implemented using a NS5000 Magnetic Stimulator (YIRUIDE Medical Corporation) with a 70‐mm‐diameter figure‐8 coil over primary motor cortex. Participants remained stationary during scalp positioning to generate maximal responses in the contralateral first dorsal interosseus. Resting motor threshold (RMT) was established as the minimum stimulation intensity inducing MEPs over 50 μV in 50% of consecutive stimulations at rest. An Visor2 neuronavigation system (ANT Neuro) was equipped to ensure stable coil positioning over the hotspot throughout the assessment.^22^

Paired‐pulse or single‐pulse TMS was stimulated in 45 trials to assess intracortical inhibition and facilitation. For each condition, 15 trials were performed with a random sequence. Details see our previous study.^23^ To evaluate active MEP and cortical silent period (CSP), participants were asked to produce a sustained submaximal (10% MVC) isometric pinch grip using the contralateral hand with stimulation intensity of 120% RMT. They were also asked to produce a constant submaximal (30% MVC) isometric pinch grip using the ipsilateral hand to assess ipsilateral silent period (iSP) with stimulation intensity of 150% RMT. During TMS testing, resting breaks were allowed. If the paretic hand could not produce measurable grip force, the active MEP, CSP, or iSP was not measured.

#### 2.5.2 TMS analysis

Custom MATLAB scripts were used to analyze MEPs offline. 15 trials of EMG data from each condition were de‐meaned and averaged. Short intracortical inhibition (SICI) was quantified by the ratio of MEP_SICI_/MEP_unconditioned_. Intracortical facilitation (ICF) was quantified using the ratio of MEP_ICF_/MEP_unconditioned._ ^23^, ^24^

### 2.6 TMS‐EEG

#### 2.6.1 TMS‐EEG recordings

TMS‐evoked EEG events were recorded using a TMS‐compatible EEG system.^18^, ^25^ Recordings were sampled at 8000 Hz with impedances maintained below 5 kΩ. TMS‐EEG recordings were performed in both hemispheres in a random order. 50 TMS pulses were applied to the primary motor cortex in each hemisphere. Stimulation was adjusted to 80% RMT. For MEP (−) individuals, RMT in the CH was used as a reference for setting stimulation intensities in the IH as well as for defining the ipsilesional motor hotspot based upon anatomical landmarks.^6^

#### 2.6.2 TMS‐EEG analysis

EEG signals were analyzed offline using custom MATLAB scripts. EEGLAB toolbox^19^ and TMSEEG toolbox^26^ were used for data preprocessing. TMS‐evoked potential (TEP), event‐related spectral perturbation (ERSP) and natural frequency in bilateral motor cortex (left: C1, C3, FC1, FC3, Cz; right: C2, C4, FC2, FC4, Cz) were calculated.^27^ Details see Data S1.

### 2.7 Statistical analysis

SPSS Statistics 22 (IBM SPSS Inc.) was used for statistical analysis. All data met the normality assumption using the Kolmogorov–Smirnov test. We separated stroke survivors into three groups based on motor function and the presence of MEPs of the paretic hand established in Session 1. MEP (−) was defined as MEP in the IH was not elicitable, and MEP (+) was defined as MEP in the IH was elicitable. Stroke survivors who were MEP (−) and had poor motor function (i.e., ARAT<10) were assigned to Group 0. Stroke survivors who were MEP (−) and had moderate‐to‐good motor function (i.e., ARAT≥10) were assigned to Group 1. Stroke survivors who were MEP (+) were assigned to Group 2. Clinical characteristics are shown in Table 1 (summary) and Table S1 (detailed).

**TABLE 1:** Patients' demographic and clinical characteristics.

	Age, years	Sex	Paretic side	Type of stroke	Lesion location	Months after stroke onset	UE FMA (0–66)	ARAT (0–57)
	Mean ± SD (range)	Male/Female	Right/Left	Ischemic/Hemorrhagic	Cortical/Subcortical/Mixed	Mean ± SD (range)	Mean ± SD (range)	Mean ± SD (range)
Group 0 (*n* = 40)	61.7 ± 11.6 (30–86)	27/13	14/26	31/9	6/28/6	4.4 ± 3.6 (1–12)	8.6 ± 5.2 (0–18)	1.7 ± 2.5 (0–10)
Group 1 (*n* = 12)	57.2 ± 13.4 (35–77)	7/5	6/6	10/2	1/8/3	3.5 ± 3.6 (1–12)	39.5 ± 12.7 (18–58)	35.3 ± 14.0 (14–55)
Group 2 (*n* = 29)	67.7 ± 8.4 (43–85)	21/8	15/14	24/5	5/15/9	3.1 ± 3.4 (1–12)	46.7 ± 15.6 (20–66)	42.8 ± 15.7 (12–57)

For baseline analysis, we used one‐way analysis of variance (ANOVA) to compare TMS, EEG, and TMS‐EEG measures among groups. Mixed design [Session (2)×Group (3)] ANOVAs were performed with repeated measures on Session. All data met the sphericity assumption using Mauchly's test. Bonferroni post hoc tests were used for multiple comparisons when *f*‐tests were significant. Pearson correlations were used between (1) baseline neurophysiological measures and subject characteristics; (2) baseline neurophysiological measures and changes in motor function between sessions; (3) changes in neurophysiological measures and changes in motor function between sessions. Statistical significance was established at *p* < 0.05. Bonferroni‐corrected *p*‐values were reported.

## 3 RESULTS

### 3.1 Cross‐sectional results

A total of 81 stroke survivors completed measurements in Session 1, including 40 subjects in Group 0, 12 subjects in Group 1, and 29 subjects in Group 2.

#### 3.1.1 EEG measures

##### EEG power

**FIGURE 1:**
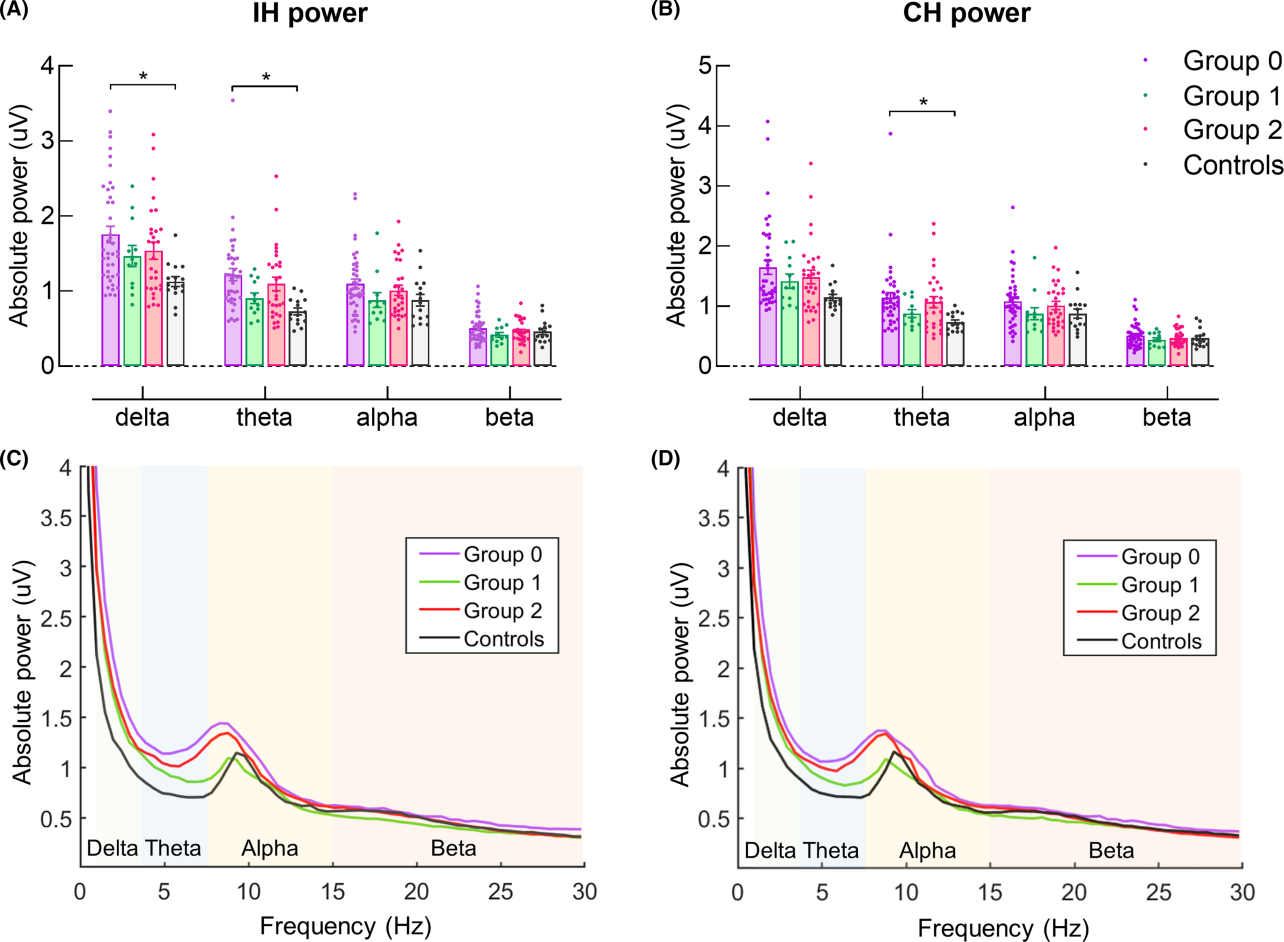
Difference in EEG power among groups. (A) IH delta and theta band power were significantly greater in Group 0 compared with healthy controls. (B) CH theta band power was significantly greater in Group 0 compared with healthy controls. (C and D) Absolute EEG power in IH and CH.

A significant main effect of Group was revealed in IH delta and theta band power (*F*_(3,92)_ = 4.20, *p* = 0.008; *F*_(3,92)_ = 5.16, *p* = 0.002, respectively) and CH theta band power (*F*_(3,92)_ = 3.36, *p* = 0.022). Post hoc comparisons revealed that for IH delta and theta bands and CH theta band, power in Group 0 was significantly greater than healthy controls (*p* = 0.004, 0.002, and 0.025, respectively) (Figure 1).

##### Interhemispheric functional connectivity

**FIGURE 2:**
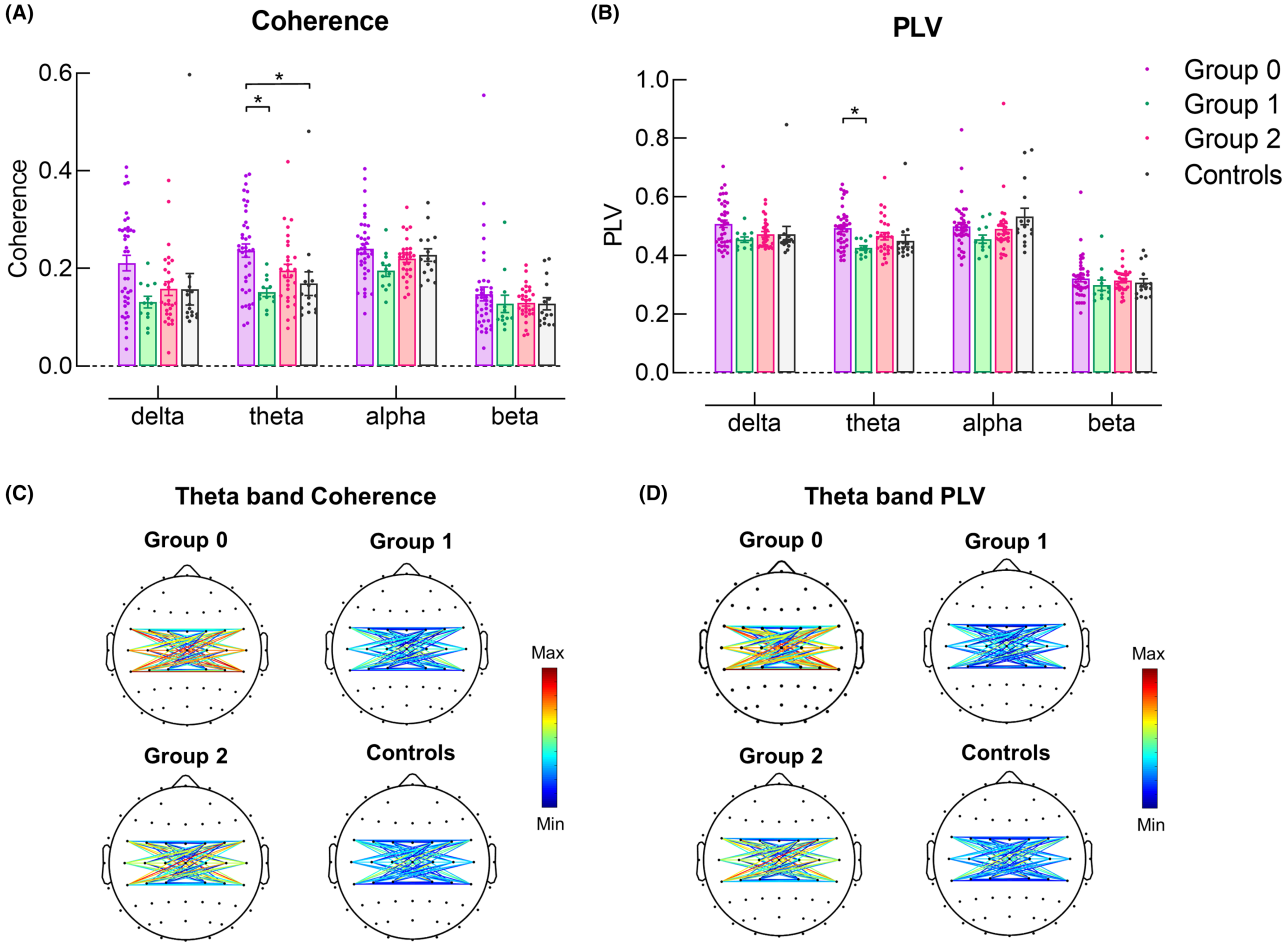
Difference in interhemispheric functional connectivity among groups. (A) Theta band coherence was significantly greater in Group 0 compared with Group 1 and healthy controls. (B) Theta band PLV was significantly greater in Group 0 compared with Group 1. *Z*‐scores of theta band coherence (C) and theta band PLV (D) between pairs of electrodes in the left and right sensorimotor cortices are presented in three stroke groups and healthy controls.

A significant main effect of Group was revealed in delta and theta band coherence and theta band PLV (*F*_(3,92)_ = 3.38, *p* = 0.022; *F*_(3,92)_ = 5.19, *p* = 0.002; *F*_(3,92)_ = 3.87, *p* = 0.012, respectively). Post hoc comparisons revealed that theta band coherence in individuals in Group 0 was significantly greater than for those in Group 1 and healthy controls (*p* = 0.008 and 0.034, respectively) (Figure 2). Theta band PLV was significantly greater in Group 0 than in Group 1 (*p* = 0.017).

##### Graph theory

A significant main effect of Group was revealed in delta band betweenness centrality (*F*_(3,92)_ = 3.10, *p* = 0.031), delta, theta, and alpha band degree centrality (*F*_(2,86)_ = 3.78, *p* = 0.013; *F*_(2,86)_ = 4.45, *p* = 0.006; *F*_(2,86)_ = 3.01, *p* = 0.034, respectively), and theta band clustering coefficient (*F*_(3,92)_ = 3.79, *p* = 0.013) in the CH. Post hoc comparisons revealed that CH delta band betweenness centrality and degree centrality, and theta band clustering coefficient were greater in Group 0 compared with Group 1 (*p* = 0.20, 0.049 and 0.049, respectively). CH theta band degree centrality was greater in Group 0 compared with Group 1 (*p* = 0.035) and healthy controls (*p* = 0.025) (Figure S1).

#### 3.1.2 TMS‐MEP measures

As TMS parameters (e.g., MEP, CSP, iSP, etc.) in the paretic hand were not recordable in MEP (−) individuals, those parameters were only compared between Group 2 and healthy controls. Paretic CSP was significantly longer in Group 2 compared with healthy controls (*p* = 0.010). TMS parameters measured in the non‐paretic hand were compared among all groups. No significant differences were observed.

#### 3.1.3 TMS‐EEG measures

**FIGURE 3:**
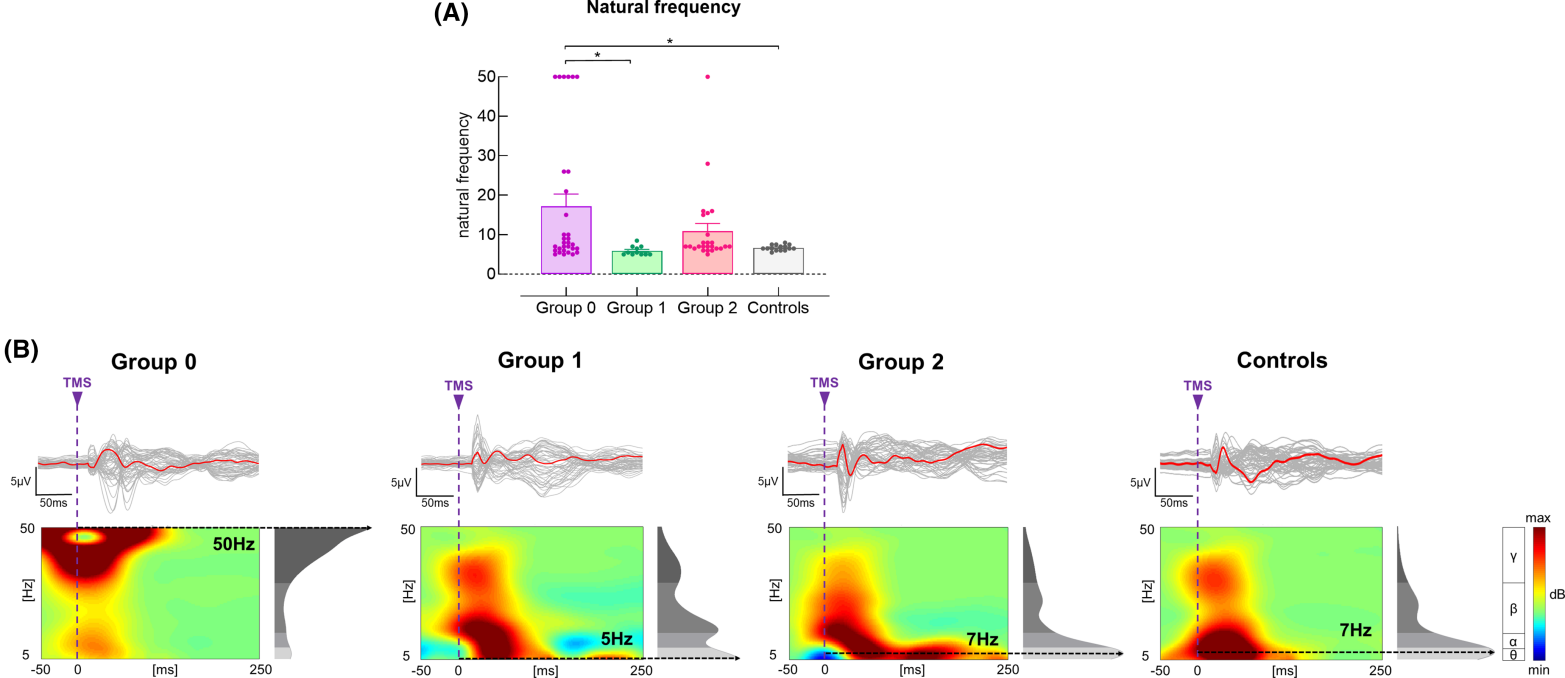
Differences in IH natural frequency among groups. (A) IH natural frequency was greater in Group 0 compared with Group 1 and healthy controls. (B) illustration of TEP, ERSP, and natural frequency in representative subjects. The gray curves represent TEP in each channel, and the red bold curves represent the averaged TEP in the channels surrounding the stimulated motor cortex. The ERSP plots show the TMS‐evoked oscillatory responses in amplitude and duration, with black doted lines highlighting the frequency with the highest power (i.e., natural frequency).

There was no significant main effect of Group revealed in any TEP component. A significant main effect of Group was revealed in IH natural frequency (*F*_(3,78)_ = 3.96, *p* = 0.011). Post hoc comparisons revealed that IH natural frequency was significantly greater in Group 0 compared with healthy controls (*p* = 0.039) and appeared to be greater compared with Group 1 (*p* = 0.052) (Figure 3).

#### 3.1.4 Correlation analysis

Negative correlations were revealed between delta band FC measures and ARAT only in Group 2 at baseline (Figure S2). For interhemispheric FC, delta band coherence and PLV were negatively correlated with ARAT (*r* ^2^ = 0.2643, *p* = 0.0043; *r* ^2^ = 0.2692, *p* = 0.0039, respectively). For FC within IH, delta band betweenness centrality and degree centrality were negatively correlated with ARAT (*r* ^2^ = 0.2141, *p* = 0.0115; *r* ^2^ = 0.2325, *p* = 0.0081, respectively). For FC in the CH, delta band degree centrality was negatively correlated with ARAT (*r* ^2^ = 0.3192, *p* = 0.0014). No significant correlation was observed between stroke chronicity and neurophysiological results (including EEG, TMS‐MEP, and TMS‐TEP measures).

### 3.2 Longitudinal results

A total of 46 stroke survivors completed measurements in Session 2: 19 in Group 0, 9 in Group 1, and 18 in Group 2.

#### 3.2.1 Behavioral measures

In all groups, FMA and ARAT were significantly higher in Session 2 than in Session 1 (*p* < 0.05). There were no significant differences between groups.

#### 3.2.2 EEG measures

##### EEG power

There was no significant difference observed in EEG power parameters.

##### Interhemispheric functional connectivity

**FIGURE 4:**
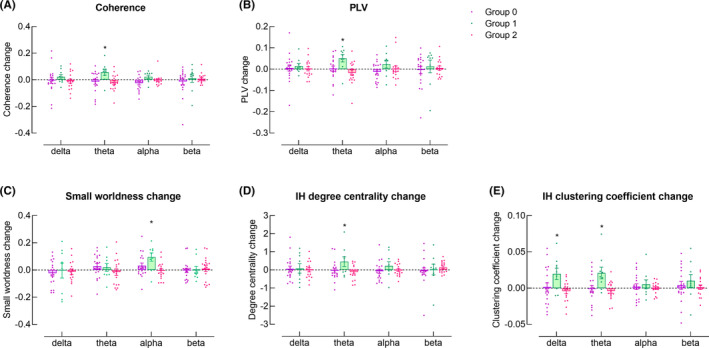
Differential changes in functional connectivity among groups. (A and B) Changes in theta band interhemispheric coherence and PLV were significantly greater in Group 1, respectively. (C) Changes in alpha band small‐worldness was significantly greater in Group 1. (D and E) Changes in IH degree centrality (theta band) and clustering coefficients (delta and theta bands) were significantly greater in Group 1, respectively.

A significant interaction of Group×Session was revealed for both theta band coherence and PLV (*F*_(2,42)_ = 3.868, *p* = 0.029; *F*_(2,42)_ = 4.679, *p* = 0.015, respectively). Post hoc comparisons revealed that in Group 1, theta band coherence and PLV were greater in Session 2 compared with Session 1 (*p* = 0.023 and 0.007, respectively) (Figure 4A,B).

##### Graph theory

Significant main effects of Session and Session×Group interaction were revealed in alpha band small‐worldness (*F*_(1,42)_ = 10.371, *p* = 0.002; *F*_(2,42)_ = 4.688, *p* = 0.015, respectively). Post hoc comparisons revealed that in Group 1, alpha band small‐worldness was significantly greater in Session 2 compared with Session 1 (*p* = 0.001) (Figure 4C).

Significant Session×Group interactions were revealed in IH theta band degree centrality and delta and theta band clustering coefficients (*F*_(2,42)_ = 3.380, *p* = 0.044; *F*_(2,42)_ = 3.551, p = 0.038; *F*_(2,42)_ = 5.683, *p* = 0.007, respectively). Post hoc comparisons suggested that the above parameters were significantly greater in Session 2 compared with Session 1 in Group 1 (*p* = 0.018, 0.010, and 0.002, respectively) (Figure 4D,E).

#### 3.2.3 TMS‐MEP measures

Among the 19 stroke survivors who were classified as Group 0 in Session 1, one subject became MEP (+) by Session 2. Among the nine stroke survivors who were classified as Group 1 in Session 1, three subjects became MEP (+) by Session 2. There was no significant difference in TMS parameters between sessions in any group or hemisphere.

#### 3.2.4 TMS‐EEG measures

There was no significant difference in TEP components or natural frequency between sessions in any group or hemisphere.

#### 3.2.5 Correlation analysis

There was a significant positive correlation between baseline alpha band PLV and ARAT change between sessions (*r* ^2^ = 0.32, *p* = 0.002) in Groups 0 and 1, but not Group 2 (Figure S3).

**FIGURE 5:**
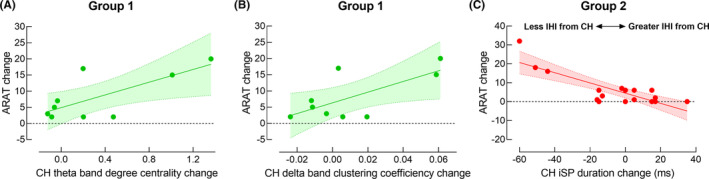
Correlations between neurophysiological change and motor function change over time. (A) There was a significant positive correlation between changes in CH theta band degree centrality and changes in ARAT score in Group 1. (B) There was a significant positive correlation between changes in CH delta band clustering coefficient and changes in ARAT score in Group 1. (C) There was a significant negative correlation between changes in paretic iSP duration and ARAT score in Group 2.

There were significant positive correlations between CH theta band degree centrality change and CH delta band clustering coefficient change and ARAT change between sessions only in Group 1 (*r* ^2^ = 0.53 and 0.48, *p* = 0.027 and 0.038, respectively) (Figure 5A,B).

There was a significant negative correlation between CH iSP change and ARAT change between sessions in Group 2 (*r* ^2^ = 0.66, *p* < 0.001) (Figure 5C).

## 4 DISCUSSION

This study for the first time combined multiple modalities of brain investigating techniques to monitor longitudinal neurophysiological changes post‐stroke. Unlike most studies that conducted analyses on a heterogeneous sample, we separated stroke survivors into three subgroups based on motor function and the presence of MEP. Our results revealed that in low‐functioning MEP (−) individuals (i.e., Group 0), FC was much higher than in other groups, and the excessive FC appeared to have a positive effect on motor recovery. In moderate‐to‐high‐functioning MEP (−) individuals (i.e., Group 1), FC increased during the course of stroke recovery and the influence from CH played a key role in motor recovery. In MEP (+) individuals (i.e., Group 2), influence from CH appeared to be detrimental to motor recovery. Our results may inform the development of new interventions for stroke rehabilitation.

### 4.1 EEG power

We observed that IH delta and theta band power and CH theta band power were higher at baseline in Group 0 than other groups. These results align with previous studies that reported enhanced low‐frequency EEG power in the IH^8^ or both hemispheres^10^, ^12^ in low‐functioning compared with higher‐functioning stroke survivors or healthy adults. Low‐frequency EEG power has been related to long‐distance communications between distant brain regions.^12^ Therefore, enhanced bilateral low‐frequency EEG power could be considered a compensatory mechanism for motor recovery in low‐functioning stroke survivors following functional or anatomical disconnections.^10^, ^12^

### 4.2 Functional connectivity

There was an increase in interhemispheric FC, FC within IH, and small‐worldness from Session 1 to Session 2 in Group 1. Some previous studies reported an increase in FC during the course of stroke recovery,^28^, ^29^ but others reported no change.^30^, ^31^ We observed an increase in FC only in Group 1, not in the whole sample, suggesting that the inconsistent results among other studies may be due to different grouping methods. These findings suggest that the dynamic changes in FC during stroke recovery are likely to be influenced by motor function and functional integrity of ipsilesional corticomotor pathway following stroke.

During stroke recovery, an association was observed in Group 1 between increases in CH FC and motor recovery, suggesting that CH supports motor recovery in these individuals. Our result is generally in line with the previous suggestion that for individuals with severe corticospinal tract damage, CH plays a supportive role in motor recovery.^4^ Furthermore, Gerloff et al.^32^ reported that well‐recovered stroke survivors had increased cortico‐cortical connectivity in motor areas of CH, indicating that CH is functionally regulated in the reformed cortical network involved in recovered hand movement post‐stroke. Therefore, support from CH could be an important recovery mechanism following stroke.

Baseline alpha band interhemispheric FC predicts motor recovery only in MEP (−) individuals, suggesting that alpha band interhemispheric FC is important for motor recovery in individuals with impaired functional integrity of the ipsilesional corticomotor pathway. Similar results have been previously reported.^33^, ^34^

### 4.3 Natural frequency

At baseline, we observed faster IH natural frequency in Group 0 compared with healthy controls. As thalamocortical neurons play a role in generating fast oscillations,^35^ increased natural frequency may reflect strengthened thalamocortical functional connections in severely impaired individuals. As thalamocortical structural connections could be disturbed due to stroke lesion‐induced cortical deafferentation,^6^ strengthened thalamocortical functional connections may compensate for structural damage and promote stroke recovery.^36^

### 4.4 Cortical and ipsilateral silent periods

As MEPs in the paretic hand were not elicitable in Groups 0 and 1, paretic CSP was only measurable in Group 2 and healthy controls. In line with previous studies that reported prolonged paretic CSP post‐stroke,^37^, ^38^ we observed that paretic CSP was longer in Group 2 compared with healthy controls. As CSP has been suggested to reflect GABA_B_ mediated intracortical inhibition processes during voluntary contractions,^5^, ^39^ prolonged paretic CSP indicates increased GABA_B_‐ergic intracortical inhibition in the IH following stroke.

We observed that paretic iSP duration was negatively correlated with motor recovery in Group 2, suggesting that individuals with greater IHI from the CH have less motor improvement during stroke recovery. A similar relationship was reported in a previous study^40^ in which paretic iSP duration was negatively associated with manual dexterity in stroke survivors. A similar correlation between IHI and motor function was also reported in an fMRI study.^41^ These findings suggest that IHI from the CH plays a negative role in motor recovery following stroke in higher‐functioning stroke survivors.

### 4.5 Clinical implications

Generally consistent with the bimodal balance recovery model in which CH is likely to play a supportive role in individuals with little structural reserve, but likely to hinder motor recovery in individuals with high structural reserve post‐stroke,^4^ our findings suggest that for MEP (−) individuals, FC (especially for those from CH) may support motor recovery, while for MEP (+) individuals, FC from CH may hinder motor recovery.

Beyond the bimodal balance recovery model, the present findings also revealed that distinct mechanisms may underlie the different dynamic changes of FC in low vs. moderate‐to‐high‐functioning MEP (−) individuals. In low‐functioning MEP (−) individuals, FC tended to be high and maintained. The excessive FC may serve as a compensatory mechanism reflecting the brain's attempt to mobilize maximal resources of the CH or other brain regions to support motor recovery. Unlike previous work suggesting that excessive FC in low‐functioning individuals is possibly ineffective,^28^ the positive correlation between baseline interhemispheric FC and motor improvement observed in the present study suggests that excessive FC is likely to play a positive role in promoting motor recovery for low‐functioning stroke survivors.

As to moderate‐to‐high‐functioning MEP (−) individuals, to our knowledge, ours is the first study to investigate these individuals as an independent subgroup. In previous studies, these individuals were usually included in MEP (−) group^8^, ^9^ or moderate‐to‐high‐functioning group,^6^ and their unique neurophysiological characteristics were often overlooked. A possible mechanism contributing to the relatively good motor function in these individuals is that alternative motor pathways may have been formed, which is further strengthened during stroke recovery presented as increased FC. The formation and strengthening of the alternative motor fibers could provide a chance for individuals with severe impairment in contralateral corticospinal tract to regain motor function. However, other factors such as low alertness during MEP testing, or unusual motor hotspot location could also cause MEPs non‐elicitable in individuals with intact contralateral corticospinal pathway. Since this mechanism alone is unable to explain the distinct neurophysiological characteristics between Groups 1 and 2, the former mechanism is more likely, and communications between brain regions is critical for motor recovery in stroke survivors with alternative motor pathway as a major recovery mechanism.

Our findings suggest that interventions for stroke recovery should be individualized rather than one‐size‐fit‐all in clinical practice. For low‐functioning MEP (−) individuals, interventions that increase FC to the IH motor area might be particularly effective. For moderate‐to‐high‐functioning MEP (−) individuals, apart from increasing FC, interventions that upregulate cortical excitability of CH motor areas might be effective. For MEP (+) individuals, interventions that inhibit the CH motor areas might be effective. Therefore, when determining which intervention to use for stroke rehabilitation, both motor function and functional integrity of the ipsilesional corticomotor pathway must be considered in order to optimize clinical efficiency.

### 4.6 Limitations

Here we used MEPs to indicate functional integrity of the ipsilesional corticomotor pathway, but fMRI was not measured, so the structural corticomotor integrity was unknown. In addition, the sample size of the present study is not very large. Further studies are needed to replicate our study with larger sample sizes.

## 5 CONCLUSIONS

This is the first study to combine multiple modalities of brain investigating techniques to monitor longitudinal neurophysiological changes post‐stroke. Our results revealed that in low‐functioning MEP (−) individuals, FC was significantly elevated, and the excessive FC appeared to have a positive effect on motor recovery. In moderate‐to‐high‐functioning MEP (−) individuals, FC increased during the course of stroke recovery and support from CH played a key role in motor recovery. In MEP (+) individuals, influence from CH appeared to be detrimental to motor recovery. These findings indicate the importance of individualized interventions for stroke rehabilitation, and for the first time suggests that both motor function and functional integrity of the ipsilesional corticomotor pathway must be considered when determining stroke rehabilitative intervention in order to optimize clinical efficiency.

## AUTHOR CONTRIBUTIONS

YL, GX, and QD designed the experiment. JiC and YP recruited the participants. QD, SC, JuC, SZ, XL, JiC, YC, and KC conducted the experiments. QD, SC, GC, and JiC reduced and analyzed the data. QD and YL interpreted the data. QD, YL, and GX wrote the manuscript.

## CONFLICT OF INTEREST STATEMENT

None declared.

## Supporting information


Data S1.

## Data Availability

The data that support the findings of this study are available on request from the corresponding author. The data are not publicly available due to privacy or ethical restrictions.
