# Supplementary material for: Neurophysiological characterization of stroke recovery: A longitudinal TMS and EEG study
Source: CNS Neurosci Ther. 2023 Sep 18;30(3):e14471. doi: 10.1111/cns.14471 (PMC10916444; doi:10.1111/cns.14471)
Supplement: Supplementary file 1 — Data S1. [file CNS-30-e14471-s001.docx]

**Supplementary Material**

**Detailed methodology for EEG**

**Data preprocessing:** The signals were down sampled to 1000 Hz, then band-pass filtered (0.1 to 40 Hz) and segmented in 2000-ms epochs. Events related to cardiac, eye (blink and movement), and muscular artifacts were excluded. In addition, trials with amplitudes over 100 μV were excluded. The resulting data were re-referenced by averaging the signals of every scalp electrode.

**EEG power calculation:** Absolute power (μV^2^) in four frequency bands [delta (1–4 Hz), theta (4–8 Hz), alpha (8–13 Hz), and beta (13–30 Hz)] was assessed by fast Fourier transform. In order to assess activity in bilateral sensorimotor cortices, the mean power of the electrodes around C3 and C4 was calculated.^1^

**Interhemispheric functional connectivity calculation**: Phase locking value (PLV), which reveal FC between bilateral sensorimotor cortices, were quantified using custom MATLAB scripts. The Welch’s averaged, modified periodogram method measures squared coherence across four frequency bands between each pair of electrodes. PLV indicates the stability of phase change between two time sequence across four frequency bands.^2^ Fisher’s z-transform was used to fit all connectivity matrices to a Gaussian distribution. The averaged z-scores from each pair of electrodes between sensorimotor cortices was used to assess interhemispheric FC.

**Graph theory analysis:** The GRaph thEoretical Network Analysis (GRETNA) toolbox was used for graph theory analysis, in which nodes and edges form the brain network.^3^ Weighted and undirected networks were built on the basis of coherence.^4^ Because there is no standard single-threshold method, we calculated the area under the curve to integrate metrics over the entire threshold range (0.1–0.4, with 0.05 interval).^3^

Global network measures include efficiency and small-worldness. Global efficiency was calculated by averaging interregional efficiency between each paired subregion, thereby characterizing the ability for information to spread within the network.^5^ Small-worldness estimates the balance between local connectedness and global integration, thus demonstrating network organization, which was quantified by the ratio of the normalized clustering coefficient value and path length.^4^

Measures of local network activity included degree centrality, betweenness centrality, and clustering coefficient. Degree centrality measures connectivity of individual nodes in a network, and high-degree nodes are structurally or functionally interconnected with other nodes.^6^ Betweenness centrality was quantified as the ratio between the number of shortest paths that pass through a given node and the total number of shortest paths in the network.^6^ The clustering coefficient was defined as the mean value of the nodal clustering coefficient (i.e., the ratio between the number of existing and possible connections in the subgraph of each given node) over all nodes. All local measures were averaged from the electrodes in motor cortex from each hemisphere.

**Detailed methodology for TMS-EEG**

**Data collection:** During TMS-EEG recordings, all participants wore inserted earplugs to avoid auditory-induced potentials as well as eye muscle reactions evoked by the TMS click.^7, 8^ A thin layer of plastic film was placed between the TMS coil and the EEG cap to minimize bone conduction produced by TMS during testing.^8, 9^

**Data preprocessing:** EEG data were sequenced around the test TMS pulse (-1000 to +1000 ms). Individual trials were corrected with the mean of the pre-stimulus baseline from -700 to -200 ms.^10, 11^ Signals from -5 to 20 ms were considered to be TMS artifacts and were discarded. Trials and channels containing high-frequency power were flagged and removed for visual inspection.^11^ In addition, TMS decay artifacts were removed based on the characteristic noise derived from independent component analysis. The trials were filtered to 1–45 Hz using a band-pass filter. Independent component analysis was implemented to remove other sources of noise, such as muscular, cardiac, or ocular artifacts.^12^ Any missing channels were linearly interpolated. All channels were referenced to the mean of all electrodes and then down sampled to 1 kHz.

**TEP calculation:** The preprocessed trials were averaged for each condition to derive TMS-evoked potential (TEP). TEP components including P30, N45, P60, N100, and P180 were calculated based on averaged TEPs for the five channels around the stimulated motor cortex.^8^

**ERSP and natural frequency calculation:** Spectral features were assessed by computing the event-related spectral perturbation (ERSP) using a Morlet wavelet transform. Evoked oscillatory response was obtained by averaging the oscillatory activity recorded in channels surrounding the stimulated motor cortex. To reduce the impact of TMS artifacts, the frequency values of the evoked oscillatory response were averaged over a 30–200 ms time window. Natural frequency was then measured as the largest cumulative ERSP frequency within 5–50 Hz upon stimulated motor cortex.^8, 13^

**Supplementary Table**

**Supplementary Table 1. Detailed demographic and clinical characteristics of stroke patients**

| **Subject number** | **Sex** | **Age** | **Months after stroke onset** | **Paretic side** | **Stroke type** | **Lesion location** | **FMA** | **Group** |
| --- | --- | --- | --- | --- | --- | --- | --- | --- |
| S01 | M | 71 | 5.8 | L | ischemic | Temporal lobe | 0 | 0 |
| S02 | F | 64 | 3.1 | R | ischemic | Frontal/parietal lobes, corona radiata | 0 | 0 |
| S03 | M | 47 | 11.5 | L | hemorrhagic | Internal capsule | 15 | 0 |
| S04 | M | 57 | 12.0 | L | hemorrhagic | Basal ganglia | 4 | 0 |
| S05 | M | 77 | 11.9 | L | ischemic | Basal ganglia, corona radiata | 22 | 2 |
| S06 | F | 60 | 1.3 | L | ischemic | Pareital lobe | 11 | 0 |
| S07 | M | 55 | 6.4 | R | ischemic | Frontal/parietal/temporal lobes | 4 | 0 |
| S08 | M | 58 | 2.7 | R | hemorrhagic | Basal ganglia | 11 | 0 |
| S09 | F | 63 | 1.2 | R | ischemic | Basal ganglia, corona radiata | 65 | 2 |
| S10 | F | 66 | 1.9 | R | ischemic | Frontal/temporal/parietal lobes and insula | 62 | 2 |
| S11 | M | 64 | 1.4 | L | ischemic | Insula, basal ganglia and corona radiata | 4 | 0 |
| S12 | F | 70 | 4.3 | L | ischemic | Insula | 15 | 0 |
| S13 | M | 73 | 11.6 | L | ischemic | Basal ganglia and corona radiata | 4 | 0 |
| S14 | M | 76 | 1.8 | L | ischemic | Basal ganglia | 0 | 0 |
| S15 | M | 61 | 2.4 | L | ischemic | Internal capsule | 66 | 2 |
| S16 | F | 69 | 3.7 | L | ischemic | Basal ganglia and corona radiata | 0 | 0 |
| S17 | F | 60 | 1.6 | R | hemorrhagic | Frontal/parietal lobes | 59 | 2 |
| S18 | M | 34 | 6.2 | L | hemorrhagic | Centrum semiovale, basal ganglia | 11 | 0 |
| S19 | M | 49 | 1.0 | L | ischemic | Corona radiata, basal ganglia | 8 | 0 |
| S20 | M | 60 | 12.0 | R | ischemic | Frontal/parietal/temporal lobes | 27 | 2 |
| S21 | F | 65 | 0.1 | R | ischemic | Corona radiata, basal ganglia | 0 | 0 |
| S22 | M | 62 | 2.6 | R | ischemic | Basal ganglia | 22 | 1 |
| S23 | M | 55 | 1.5 | L | ischemic | Centrum semiovale, corona radiata, basal ganglia, pons | 4 | 0 |
| S24 | M | 71 | 11.3 | L | ischemic | Centrum semiovale, corona radiata, basal ganglia | 16 | 0 |
| S25 | M | 72 | 0.8 | L | ischemic | Lateral ventricle, internal capsule | 52 | 2 |
| S26 | M | 71 | 0.7 | L | hemorrhagic | Parietal/temporal lobes, basal ganglia | 35 | 2 |
| S27 | M | 65 | 1.7 | R | ischemic | Basal ganglia, corona radiata, pons | 57 | 2 |
| S28 | F | 30 | 5.3 | L | hemorrhagic | Parietal/temporal lobes, lateral ventricle | 12 | 0 |
| S29 | M | 45 | 4.5 | R | hemorrhagic | Centrum semiovale, corona radiata, basal ganglia | 0 | 0 |
| S30 | M | 51 | 0.3 | R | ischemic | Centrum semiovale, corona radiata, basal ganglia, insula | 44 | 1 |
| S31 | M | 68 | 11.9 | R | ischemic | Basal ganglia | 14 | 0 |
| S32 | M | 52 | 4.4 | L | ischemic | Temporal/occipital/frontal lobes, insula, basal ganglia | 4 | 0 |
| S33 | F | 63 | 0.4 | R | ischemic | Corona radiata, basal ganglia | 46 | 1 |
| S34 | F | 49 | 2.1 | L | ischemic | Corona radiata, basal ganglia | 51 | 1 |
| S35 | M | 67 | 1.4 | L | ischemic | Internal capsule, insula | 66 | 2 |
| S36 | M | 83 | 11.0 | R | ischemic | Parietal/temporal/occipital/frontal lobes, corona radiata, basal ganglia | 31 | 2 |
| S37 | M | 86 | 3.1 | L | ischemic | Corona radiata, basal ganglia | 13 | 0 |
| S38 | M | 64 | 0.5 | R | ischemic | Frontal/parietal/temporal lobes | 58 | 2 |
| S39 | F | 72 | 1.4 | R | ischemic | Centrum semiovale, corona radiata | 4 | 0 |
| S40 | F | 64 | 1.4 | R | ischemic | Frontal/parietal/temporal lobes | 14 | 0 |
| S41 | M | 70 | 7.4 | L | ischemic | Pons | 37 | 2 |
| S42 | M | 57 | 9.0 | R | hemorrhagic | Centrum semiovale, corona radiata | 47 | 2 |
| S43 | F | 59 | 2.6 | L | ischemic | Basal ganglia | 9 | 0 |
| S44 | M | 70 | 1.7 | L | ischemic | Corona radiata, basal ganglia | 14 | 0 |
| S45 | F | 85 | 1.5 | L | ischemic | Basal ganglia | 62 | 2 |
| S46 | F | 66 | 1.4 | L | ischemic | Internal capsule, basal ganglia | 4 | 0 |
| S47 | M | 77 | 0.9 | L | ischemic | Centrum semiovale, corona radiata, basal ganglia | 58 | 1 |
| S48 | F | 35 | 4.9 | L | ischemic | Frontal/parietal lobes, corona radiata | 49 | 1 |
| S49 | M | 69 | 9.7 | R | ischemic | Basal ganglia | 18 | 1 |
| S50 | F | 81 | 0.7 | R | ischemic | Basal ganglia | 23 | 2 |
| S51 | F | 65 | 3.1 | L | hemorrhagic | Basal ganglia, thalamus | 47 | 2 |
| S52 | M | 46 | 5.0 | L | hemorrhagic | Lateral ventricle | 27 | 1 |
| S53 | M | 70 | 1.5 | R | ischemic | Basal ganglia | 37 | 2 |
| S54 | M | 63 | 1.9 | R | hemorrhagic | Basal ganglia, frontal lobe | 10 | 0 |
| S55 | M | 72 | 2.1 | R | ischemic | Parietal/temporal lobes, lateral ventricle | 53 | 2 |
| S56 | M | 62 | 1.9 | L | ischemic | Pons | 35 | 2 |
| S57 | M | 63 | 6.9 | L | ischemic | Centrum semiovale, corona radiata, basal ganglia, cerebellum | 12 | 0 |
| S58 | F | 70 | 1.8 | L | ischemic | Corona radiata, basal ganglia | 45 | 1 |
| S59 | M | 75 | 4.5 | L | ischemic | Pons | 5 | 0 |
| S60 | M | 35 | 12.0 | L | hemorrhagic | Basal ganglia | 24 | 1 |
| S61 | M | 67 | 1.2 | L | ischemic | Frontal/parietal/temporal lobes | 4 | 0 |
| S62 | F | 54 | 0.3 | L | ischemic | Corona radiata | 10 | 0 |
| S63 | M | 64 | 0.6 | L | ischemic | Centrum semiovale, corona radiata, basal ganglia, thalamus | 25 | 2 |
| S64 | M | 42 | 5.8 | L | hemorrhagic | Thalamus/occipital lobes | 21 | 0 |
| S65 | F | 79 | 2.1 | R | ischemic | Frontal/parietal/temporal lobes | 36 | 2 |
| S66 | M | 60 | 8.8 | R | ischemic | Frontal/parietal lobes, basal ganglia, corona radiata | 4 | 0 |
| S67 | M | 43 | 0.9 | L | ischemic | Parietal/temporal lobes | 64 | 2 |
| S68 | M | 68 | 3.8 | R | ischemic | Internal capsule | 23 | 0 |
| S69 | F | 64 | 3.1 | R | hemorrhagic | Basal ganglia, parietal lobe | 20 | 2 |
| S70 | M | 64 | 1.4 | L | ischemic | Corona radiata, basal ganglia, thalamus | 49 | 2 |
| S71 | M | 75 | 12.0 | L | ischemic | Frontal/parietal/temporal lobes, basal ganglia | 9 | 0 |
| S72 | F | 58 | 0.7 | R | ischemic | Parietal/temporal lobes | 42 | 1 |
| S73 | M | 64 | 1.8 | L | ischemic | Frontal/parietal/temporal/occipital lobes, centrum semiovale, corona radiata, basal ganglia | 28 | 2 |
| S74 | M | 68 | 3.2 | L | ischemic | Basal ganglia, corona radiata, centrum semiovale, frontal lobe | 66 | 2 |
| S75 | M | 76 | 2.7 | R | hemorrhagic | Basal ganglia | 12 | 0 |
| S76 | M | 73 | 0.8 | R | ischemic | Thalamus | 66 | 2 |
| S77 | M | 72 | 2.7 | R | ischemic | Frontal/parietal lobes, corona radiata, centrum semiovale, lateral ventricle | 57 | 2 |
| S78 | M | 63 | 1.0 | L | ischemic | Basal ganglia | 18 | 0 |
| S79 | F | 49 | 0.9 | R | ischemic | Pons | 23 | 0 |
| S80 | M | 71 | 2.1 | R | ischemic | Centrum semiovale, lateral ventricle, basal ganglia, frontal lobe, insula | 49 | 1 |
| S81 | F | 62 | 3.9 | R | ischemic | Internal capsule, basal ganglia | 5 | 0 |

**Notes:** M stands for male, and F stands for female. L stands for left, and R stands for right.

**Supplementary Figures**

**Supplementary Figure 1:** Difference in CH functional connectivity among groups. **(A)** Theta band betweenness centrality was significantly greater in Group 0 compared with Group 1. **(B)** Delta band degree centrality was significantly greater in Group 0 compared with Group 1. Theta band degree centrality was significantly greater in Group 0 compared with Group 1 and healthy controls. **(C)** Theta band clustering coefficient was significantly greater in Group 0 compared with healthy controls. Data presented are group mean ± standard error.


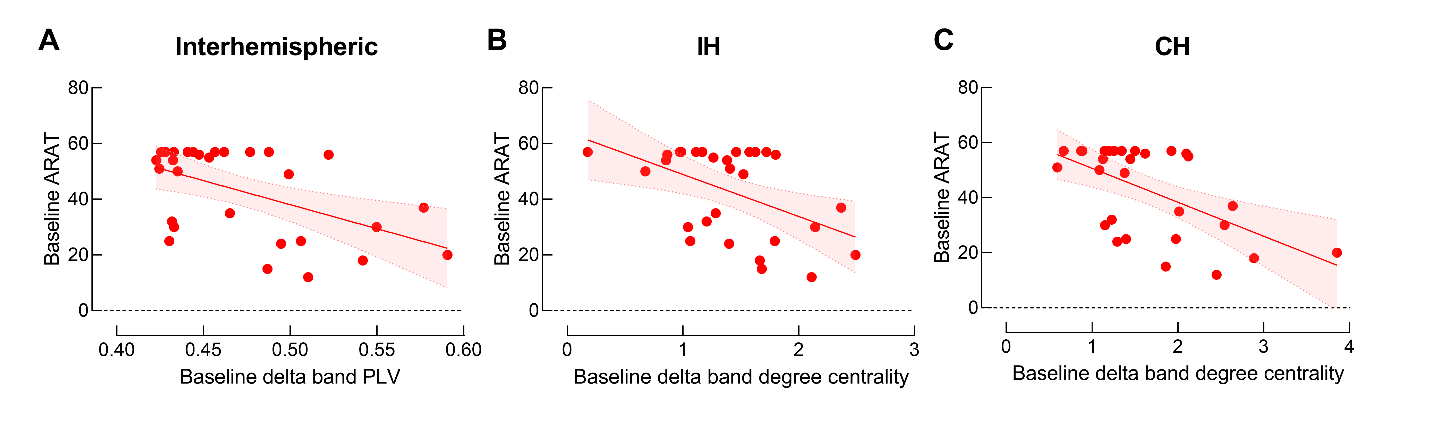


**Supplementary Figure 2: Correlations between baseline functional connectivity and motor function in Group 2. (A)** There was significant negative correlation between baseline delta band interhemispheric PLV and ARAT. **(B and C)** There were significant negative correlations between baseline ARAT and delta band degree centrality in both hemispheres.

**
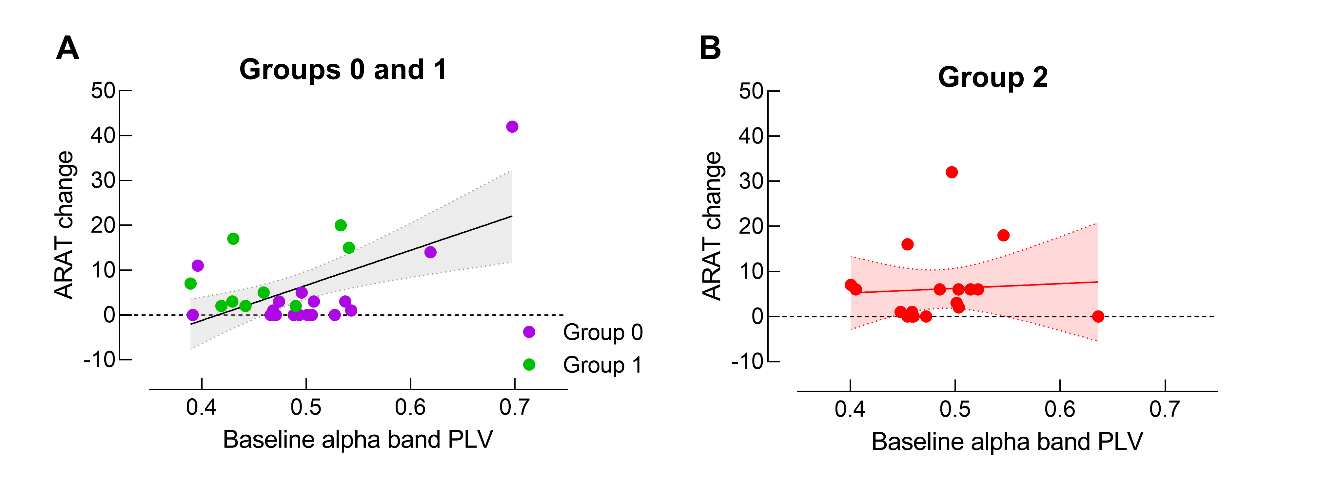
**

**Supplementary Figure 3: Correlations between baseline alpha band interhemispheric functional connectivity and changes in motor function over time.** There was a significant positive correlation between baseline alpha band interhemispheric PLV and changes in ARAT in Groups 0 and 1 (A), but not in Group 2 (B).

**References**

1. Bayram MB, Siemionow V, Yue GH. Weakening of Corticomuscular Signal Coupling During Voluntary Motor Action in Aging. J Gerontol A Biol Sci Med Sci. 2015 Aug;70(8):1037-43.

2. Briels CT, Schoonhoven DN, Stam CJ, et al. Reproducibility of EEG functional connectivity in Alzheimer's disease. Alzheimers Res Ther. 2020 Jun 3;12(1):68.

3. Wang J, Wang X, Xia M, et al. GRETNA: a graph theoretical network analysis toolbox for imaging connectomics. Frontiers in human neuroscience. 2015;9:386.

4. Vecchio F, Tomino C, Miraglia F, et al. Cortical connectivity from EEG data in acute stroke: A study via graph theory as a potential biomarker for functional recovery. Int J Psychophysiol. 2019 Dec;146:133-8.

5. Park CH, Chang WH, Yoo WK, et al. Brain topological correlates of motor performance changes after repetitive transcranial magnetic stimulation. Brain Connect. 2014 May;4(4):265-72.

6. Rubinov M, Sporns O. Complex network measures of brain connectivity: uses and interpretations. NeuroImage. 2010 Sep;52(3):1059-69.

7. ter Braack EM, de Vos CC, van Putten MJ. Masking the Auditory Evoked Potential in TMS-EEG: A Comparison of Various Methods. Brain Topogr. 2015 May;28(3):520-8.

8. Tscherpel C, Dern S, Hensel L, et al. Brain responsivity provides an individual readout for motor recovery after stroke. Brain. 2020 Jun 1;143(6):1873-88.

9. Massimini M, Ferrarelli F, Esser SK, et al. Triggering sleep slow waves by transcranial magnetic stimulation. Proc Natl Acad Sci U S A. 2007 May 15;104(20):8496-501.

10. Atluri S, Frehlich M, Mei Y, et al. TMSEEG: A MATLAB-Based Graphical User Interface for Processing Electrophysiological Signals during Transcranial Magnetic Stimulation. Front Neural Circuits. 2016;10:78.

11. Sun Y, Blumberger DM, Mulsant BH, et al. Magnetic seizure therapy reduces suicidal ideation and produces neuroplasticity in treatment-resistant depression. Translational psychiatry. 2018 Nov 23;8(1):253.

12. Delorme A, Sejnowski T, Makeig S. Enhanced detection of artifacts in EEG data using higher-order statistics and independent component analysis. Neuroimage. 2007 Feb 15;34(4):1443-9.

13. Tremblay S, Rogasch NC, Premoli I, et al. Clinical utility and prospective of TMS-EEG. Clin Neurophysiol. 2019 May;130(5):802-44.
